# 

**Published:** 1831-01

**Authors:** 


### LONDON MEDICAL AND PHYSICAL JOURNAL.

EDITED BY

JOHN NORTH, ESQ. F.L.S.

MEMBER OF THE ROYAL COLLEGE OF SURGEONS,

AND OF THE MEDICAL AND CHIRURGICAL SOCIETY OF LONDON.

---

(VOL. LXVI.)

NEW SERIES, VOL. X.

---

Et quouiam variant morbi, variabimus artes;

Mille mali species, mille salutis erunt.

---

INDEXED S.G.O.

Surgeon Gen'l's Office LIBRARY

45-5-98

LONDON:

JOHN SOUTER, 73, ST. PAUL'S CHURCH-YARD;

AND TO BE HAD OF ALL MEDICAL BOOKSELLERS.

1831.

W1
WO 31

J. AND C. ADLARD, PRINTERS,

BARTHOLOMEW CLOSE.

### MONTHLY LIST OF MEDICAL BOOKS.

[*Medical Works cannot be entered on this List unless a copy be sent for the purpose, the titles of Books having frequently been sent to us as published, which have not been published for weeks, or even months, after.*]

Illustrations of Mr. S. Cooper's Surgical Dictionary. Published monthly. Each Part containing four Lithographic Plates, with Letter-press Descriptions, and References to the Text. Parts I. II. III. and IV.—London: Longman, 1830.

These Illustrations cannot fail to be of the greatest assistance to the surgical student. The object is to give a faithful delineation of all the diseases mentioned in that well-known and most excellent work, the Surgical Dictionary. Many original sketches are also to be given of diseased parts in the various museums in the metropolis. At the bottom of each page of the descriptive letter-press, the page of the Dictionary is referred to, in which an account of the disease, illustrated, may be found. A similar undertaking has been well received in Germany, and we have no doubt of its perfect success in this country. The plates in the three first parts are very neatly and correctly executed.

A New Mode of Ventilating Hospitals, Ships, Prisons, &c.: being an efficient Method of destroying Contagion, and of preventing the spreading of Infectious Diseases. By George Hawthorne, m.d.—8vo. Belfast, 1830. London: Longman.

An Introductory Lecture to the Theory and Practice of Midwifery; being an Historical Account of that subject. Publicly delivered in London, October 4th, 1829. By Thomas Greening, m.d. late Obstetric Physician to the City of London Lying-in Charity, &c. Second Edition.—4to. pp. 39. London: Limebeer, 1831.

Elements of Pathology, and Practice of Physic. By John Mackintosh, m.d. Acting Surgeon to the Ordnance in North Britain, Lecturer on the Practice of Physic in Edinburgh, &c. Vol. I.—8vo. pp. 484. London: Longman.

Syllabus of Lectures on Medical Jurisprudence in the University of London. By A. T. Thomson, m.d. f.l.s. Professor of Materia Medica and Therapeutics; and Andrew Amos, Esq. a.m. Barrister, Professor of Law.—8vo. pp. 15. London: Mallett, 1830.

Examen des Opinions de M. le Docteur Castel, touchant la prĕtendue Contagion de la Fièvre Jaune; ou, Reponse à un écrit intitulé, De la Contagion dans les Affections Fébriles. Par N. Chervin, m.d.p. &c.—8vo. pp. 51. London: Baillière, 1830.

Anatomical Demonstrations; or, Colossal Illustrations of Human Anatomy. By Professor Seerig. Translated from the German. Part I.—Schloss, Foreign Bookseller, Southampton buildings, Chancery lane. 1831.

The great difficulty of clearly making out the various parts in anatomical plates of a small size, must often perplex the student. Professor Seerig has, therefore, very judiciously facilitated the study of anatomy, by the publication of these anatomical illustrations on a scale larger than life. But one part of this very useful work has yet appeared. It consists of four plates: the first is copied from the elder Meckel's plate of the nerves of the face; the second, with the exception of some alterations made by Professor Seerig, is taken from the second plate of Bock's Dissection of the Fifth Cerebral Nerve; the third and fourth plates are chiefly derived from Soemmerring's works on the ear and eye. The work will consist of six parts, each of which will appear at short intervals until published. In the list of subscribers, we observe the names of many of our first surgical and anatomical professors. The price of these "Demonstrations," when complete, will be very moderate.

Patterson's *Portable Consommé.*

This composition is exactly to our taste: we finished it in a few minutes. It is not a volume of erudition, but a *pot* of strong animalised jelly, which, when mixed with water, makes a basin of most excellent soup. Proper directions are enclosed with the pots, which contain, according to the prices affixed to them, sufficient for a basin of half a pint, to that of a tureen of two quarts. The Consommé will keep for years: it will be found a very good, cheap, and convenient article. As a proof of our opinion of the merit of this *work*, we beg to assure the proprietor that we shall go through a second or third edition with much pleasure!

